# Evolutionary Roots and Diversification of the Genus *Aeromonas*

**DOI:** 10.3389/fmicb.2017.00127

**Published:** 2017-02-08

**Authors:** Ariadna Sanglas, Vicenta Albarral, Maribel Farfán, J. G. Lorén, M. C. Fusté

**Affiliations:** ^1^Departament de Biologia, Sanitat i Medi Ambient, Secció de Microbiologia, Facultat de Farmàcia i Ciències de l’Alimentació, Universitat de BarcelonaBarcelona, Spain; ^2^Institut de Recerca de la Biodiversitat, Universitat de BarcelonaBarcelona, Spain

**Keywords:** *Aeromonas*, *mdh*, *recA*, chronogram, diversification model, divergence time

## Abstract

Despite the importance of diversification rates in the study of prokaryote evolution, they have not been quantitatively assessed for the majority of microorganism taxa. The investigation of evolutionary patterns in prokaryotes constitutes a challenge due to a very scarce fossil record, limited morphological differentiation and frequently complex taxonomic relationships, which make even species recognition difficult. Although the speciation models and speciation rates in eukaryotes have traditionally been established by analyzing the fossil record data, this is frequently incomplete, and not always available. More recently, several methods based on molecular sequence data have been developed to estimate speciation and extinction rates from phylogenies reconstructed from contemporary taxa. In this work, we determined the divergence time and temporal diversification of the genus *Aeromonas* by applying these methods widely used with eukaryotic taxa. Our analysis involved 150 *Aeromonas* strains using the concatenated sequences of two housekeeping genes (approximately 2,000 bp). Dating and diversification model analyses were performed using two different approaches: obtaining the consensus sequence from the concatenated sequences corresponding to all the strains belonging to the same species, or generating the species tree from multiple alignments of each gene. We used BEAST to perform a Bayesian analysis to estimate both the phylogeny and the divergence times. A global molecular clock cannot be assumed for any gene. From the chronograms obtained, we carried out a diversification analysis using several approaches. The results suggest that the genus *Aeromonas* began to diverge approximately 250 millions of years (Ma) ago. All methods used to determine *Aeromonas* diversification gave similar results, suggesting that the speciation process in this bacterial genus followed a rate-constant (Yule) diversification model, although there is a small probability that a slight deceleration occurred in recent times. We also determined the constant of diversification (λ) values, which in all cases were very similar, about 0.01 species/Ma, a value clearly lower than those described for different eukaryotes.

## Introduction

Prokaryotes are an essential and largely unnoticed component of the earth’s biota. They play a crucial role in all biogeochemical cycles of the biosphere and produce important components of the earth’s atmosphere. Although prokaryotes represent the majority of the global biomass of living organisms, and dominated the first 80% of the history of life, the macroevolutionary models established for eukaryotes have been scarcely applied to them (Martin et al., 2004), and the origin of a bacterial lineage or the way in which it has diversified remains largely unexplored. There are only a few references in the literature about bacterial diversification (Martin et al., 2004; Vinuesa et al., 2005; Barraclough et al., 2009; Morlon et al., 2012; Lorén et al., 2014), and in no case has the reported analysis been as complete as those published on higher organisms. Despite the importance of diversification rates in the study of prokaryote evolution, they have not been quantitatively assessed for the majority of microorganism taxa. The investigation of evolutionary patterns in prokaryotes constitutes a challenge, due to the absence of a reliable fossil record, limited morphological differentiation and frequently complex taxonomic relationships.

Since Nee et al. (1994) proposed a method to estimate both speciation and extinction rates of a lineage from phylogenies reconstructed from contemporary taxa, several other methods mainly based on birth–death models have been developed (Sanderson and Donoghue, 1996; Aldous, 2001; Nee, 2006). In the simplest of these models, the birth and death rates of lineages remain constant through time. However, rates of species origination and extinction can vary over time during evolutionary radiations and among lineages (Rabosky and Lovette, 2008; Morlon et al., 2010). Therefore, several authors have developed methods to estimate changes in diversification rates through time and across lineages from phylogenetic data of extant species (Nee et al., 1994; Paradis et al., 2004; Rabosky, 2009). All these methods have potential applications in the study of speciation and extinction processes in organisms with few or non-existent fossil records, such as prokaryotes, although a major problem is the difficulty in estimating divergence times. Phylogenetic trees derived from DNA sequences only contain information about the relative timing of reconstructed speciation events. The units of branch length are usually nucleotide substitutions per site, that is, the number of changes or ‘substitutions’ divided by the length of the sequence. The branch lengths (not the nodes) in some trees (dated trees) may be interpreted as time estimates. When building a tree, every reconstruction method gives a branch length (bl), which is a function of the rate of substitution (μ) and the time of evolution (t): bl = μt. To estimate the divergence time t of each node, it is necessary to separate the two parameters in each branch, modeling how μ might vary between every branch in the tree. After obtaining μ, it is easy to calculate t (t = bl/μ). This will give a relative time scale. To convert the relative into absolute divergence times it is necessary to have external information on the absolute dates of one or more nodes in the tree. This can be achieved by imposing constraints on some interior nodes, such as fossils, geological events or other indirect evidence.

Following the publications of Zuckerkandl and Pauling (1965) and Kimura (1968), molecular dating has been based on the molecular clock hypothesis of a constant chronological rate of sequence change (Lemey and Posada, 2009). This approach has been regularly challenged by results obtained using datasets from a variety of organisms, ranging from bacteria to primates, which show considerable departures from clocklike evolution and constant rate variation among lineages, and it has become clear that the strict molecular clock hypothesis is not biologically realistic (Drummond et al., 2006). This implies that although it is possible to infer phylogenies from molecular sequences, it is not possible to estimate molecular rates or divergence times, because the individual contribution of each one to molecular evolution cannot be separated (Felsenstein, 1981; Drummond et al., 2006; Lepage et al., 2007).

Among the challenges associated with the study of macroevolutionary patterns in microorganisms, one of the most significant is to determine if the diversification rate is constant or varies over time. The limited studies in this field have been mainly based on pathogenic bacteria, in which diversification rates seem not to be constant (Morlon et al., 2012). Controversially, the very few studies on free-living or symbiotic bacteria suggest a constant rate of diversification (Martin et al., 2004; Vinuesa et al., 2005).

The genus *Aeromonas* Stanier 1943 (Martin-Carnahan and Joseph, 2005) is a *Gammaproteobacteria* (*Proteobacteria, Bacteria*) that comprises a group of Gram-negative, rod-shaped bacteria, which are autochthonous to aquatic environments worldwide and are usual microbiota (as well as primary or secondary pathogens) of fish, amphibians and other animals (Janda and Abbott, 2010). Some species, mainly *A. caviae. A. hydrophila* and *A. veronii* bv. Sobria, are opportunistic pathogens of humans, in which they produce diseases with a broad severity spectrum, ranging from mild diarrhea to life-threatening infections (Janda and Abbott, 2010; Parker and Shaw, 2011). Hence, the *Aeromonas* genus constitutes a perfect scenario to study the diversification processes in bacteria due to the huge variety of habitats from which its species can be isolated and its combination of free-living bacteria and host-associated strains.

The main objective of this work is to determine the divergence time and the pattern of diversification of *Aeromonas* from phylogenetic data obtained applying Bayesian reconstructions. The phylogeny was constructed from the sequences of two housekeeping genes determined in 150 strains corresponding to the different species of this bacterial genus. We used the divergence time of *Escherichia coli* and *Salmonella enterica* as the calibration point. Molecular dating and macro evolutionary birth–death models were used to determine the temporal pattern of lineage diversification and significant changes in diversification rates were estimated using models with constant and variable diversification rates (Rabosky, 2006).

## Materials and Methods

### Gene Sequences

A collection of 150 *Aeromonas* strains, representative of the 27 species recognized up to August 2015, was selected for the study. Bacterial isolates and reference strains were obtained from several type culture collections, kindly supplied by other authors (Katri Berg, University of Helsinki, Helsinki, Finlandia; Yogesh Shouche, Molecular Biology Laboratory, National Centre for Cell Science, Pune, India; Margarita Gomila, Universitat de les Illes Balears, Palma de Mallorca, Spain; M^a^ José Figueras, Universitat Rovira i Virgili, Reus, Spain; Antonio Martínez-Murcia, Universidad de Alicante, Spain) or from samplings of freshwater and food carried out by our research group (Miñana-Galbis et al., 2002). Species-level identification of these isolates were performed in previous studies by phenotypical and/or molecular approaches (Miñana-Galbis et al., 2002; Miñana-Galbis et al., 2009; Farfán et al., 2010; Fusté et al., 2012; Sanglas et al., 2016). Bacterial culture conditions and genomic DNA extraction were performed as described previously (Farfán et al., 2010). Two housekeeping genes (*mdh* and *recA*) were chosen for the analysis; for each strain, the full-length sequences for both genes were obtained, using methods previously reported (Farfán et al., 2010; Sanglas, 2015; Sanglas et al., 2016). The sequences determined in this paper were deposited in the GenBank^1^. The strains and sequences used in this study are listed in **Supplementary Table S1**, indicating the species affiliation, source and geographical origin of these isolates and the GenBank accession numbers of the gene sequences.

### Data Sets

Phylogenetic reconstruction of all strains was carried out from the concatenated sequences of *mdh* and *recA* genes. For each gene, the translated sequences were aligned using the ClustalW program (Thompson et al., 1994) implemented in MEGA6 (Tamura et al., 2013) and translated back to obtain the nucleotide alignments. Both alignments were concatenated with the DAMBE program (v5.3.10; Xia, 2013).

Dating and diversification model analyses were performed using two different approaches to obtain one sequence per species. In one approach, the consensus sequence for each species was obtained from the sequences of all the strains belonging to the same species. For those species with only a single strain, the concatenated sequence was used. The consensus DNA sequences were obtained using the R seqinr package (Charif and Lobry, 2007) and the majority method option, in which the character with the highest frequency is returned as the consensus character. In the second approach, we generated the species tree from multiple alignments of each gene as separate data partitions, with several individuals per species, using the starBEAST method (Heled and Drummond, 2010), an extension of the BEAST (Bayesian Evolutionary Analysis Sampling Trees) software package (Drummond and Rambaut, 2007).

### Phylogenetic Analysis

Bayesian phylogenetic trees were reconstructed with the BEAST program (v1.8.1; Drummond et al., 2012) from the data sets. The model of evolution for each gene was determined using the jModelTest 2 program (Darriba et al., 2012). The general time-reversible model with discrete gamma distribution and invariant sites (GTR+G+I) was selected as the best-fit model of nucleotide substitution. The Bayesian analyses were performed using a GTR model with four gamma categories, a Yule process of speciation, and an uncorrelated lognormal relaxed-clock model of rate as the tree priors, as well as other default parameters. We performed three independent Markov Chain Monte Carlo (MCMC) runs of 20 (consensus tree), 50 (all strains) or 100 (species tree) million generations, sampling every 2,000 (consensus tree) or 5,000 (all strains and species tree) generations. Posterior distributions for parameter estimates and likelihood scores to approximate convergence were visualized with the Tracer program (v1.6.0; Rambaut et al., 2014). Visual inspection of traces within and across runs, as well as the effective sample sizes (EES) of each parameter (>200), allowed us to confirm that the analyses were adequately sampled. A maximum clade credibility (MCC) tree was chosen by TreeAnnotator (v1.8.1; Drummond et al., 2012) from the combined output of the three MCMC runs using the LogCombiner program^2^ after the removal of the initial trees (20–25%) as burn-in. The MCC tree was visualized with the program FigTree^3^ (v1.4.2).

### Divergence Time Estimations

We generated the consensus and species trees by Bayesian inference. Molecular dating was determined using BEAST, simultaneously estimating both the phylogeny and the divergence times from the corresponding chronogram (ultrametric tree). We used the divergence time between *E. coli* and *S. enterica* estimated by Ochman and Wilson as the calibration point (Ochman and Wilson, 1987a,b). Accordingly, we calibrated the divergence of *Aeromonas* with a normally distributed prior with a mean of 140 Ma and a standard deviation of 10 Ma. For all dating analyses, we applied a Bayesian relaxed-clock approach, implemented in BEAST, with an uncorrelated lognormal clock model that assumes an underlying lognormal distribution (UCLD) of the evolutionary rates. This relaxed-clock method can account for a rate heterogeneity across lineages and accommodate multiple calibrations. Moreover, it can incorporate multiple loci into one analysis and deal appropriately with different rates among loci.

### Diversification Analyses

All analyses were performed in the R environment (v3.1.3; R Core Team, 2016) using functions implemented in ape (Paradis et al., 2004), LASER (Rabosky, 2006, 2009) and TreeSim (Stadler, 2011) packages. MCC ultrametric trees (consensus and species tree chronograms) were used after excluding the calibration outgroup.

Standard lineages-through-time (LTT) plots, linear regression analysis, and LTT plots obtained from 1,000 simulated phylogenies with the same size and diversification rate for each set were generated as previously described (Lorén et al., 2014), to graphically visualize and evaluate the temporal pattern of lineage diversification in *Aeromonas*. Moreover, we also estimated the theoretical LTT curve, a method recently developed by Paradis (2015), to assess the fit of our data.

We used the birth–death likelihood (BDL) tests implemented in LASER to detect the temporal pattern of diversification and the speciation and extinction rates (λ and μ) from the *Aeromonas* phylogeny. The LTT plot derived from the MCC tree was used to test the null hypothesis of no-rate change versus variable-rate change in diversification, applying the maximum likelihood (ML) approach of Rabosky, the test ΔAIC_RC_ (Rabosky, 2009). This statistic is calculated as: ΔAIC_RC_ = AIC_RC_ - AIC_RV_, where AIC_RC_ is the Akaike information criterion (AIC) score for the best fitting rate-constant diversification model, and AIC_RV_ is the AIC for the best fitting variable-rate diversification model. Thus, a positive value for ΔAIC_RC_ indicates that the data are best approximated with a rate-variable model, while a negative ΔAIC_RC_ value suggests a rate-constant model of diversification. We tested five different models, two of which were rate-constant (pure-birth or Yule and birth–death) and three were rate-variable (DDL, DDX and Yule 2-rates) (Lorén et al., 2014).

We calculated the gamma (γ) statistic (Pybus and Harvey, 2000) and its significance by simulating 1,000 phylogenies, as described previously (Lorén et al., 2014). This statistic compares the relative node positions in a phylogeny with those expected under a constant diversification rate model, in which the statistic follows a standard normal distribution. Positive γ values evidence that nodes are closer to the tips than expected under the constant rate model. When γ is negative, the internal nodes are closer to the root than expected under a constant model, indicating a decrease in diversification through time. In addition, we compared the observed empirical gamma value with the gamma distribution obtained by simulation.

Finally, in order to detect variations in evolutionary rates through time and among lineages, we used the BAMM (Bayesian Analysis of Macroevolutionary Mixtures) program (Rabosky, 2014^4^). All the results and calculations were visualized using the BAMMtools package (Rabosky et al., 2014), from which we obtained a phylogenetic tree with the diversification rates in each branch, as well as the net diversification rates through time. Moreover, we estimated the cumulative probabilities of the number of rate shifts in a phylogeny (models with 0, 1 or several shifts) and the Bayes factor (BF). The BF (Kass and Raftery, 1995) is the ratio of the posterior probabilities of two models: a model with zero rate shifts and another with at least one diversification shift. The BF criterion is not worthy (1–3.2), moderate (3.2–10), strong (10–100), or decisive (>100) evidence in favor of the numerator model.

## Results

### Phylogenetic Analysis

**FIGURE 1:**
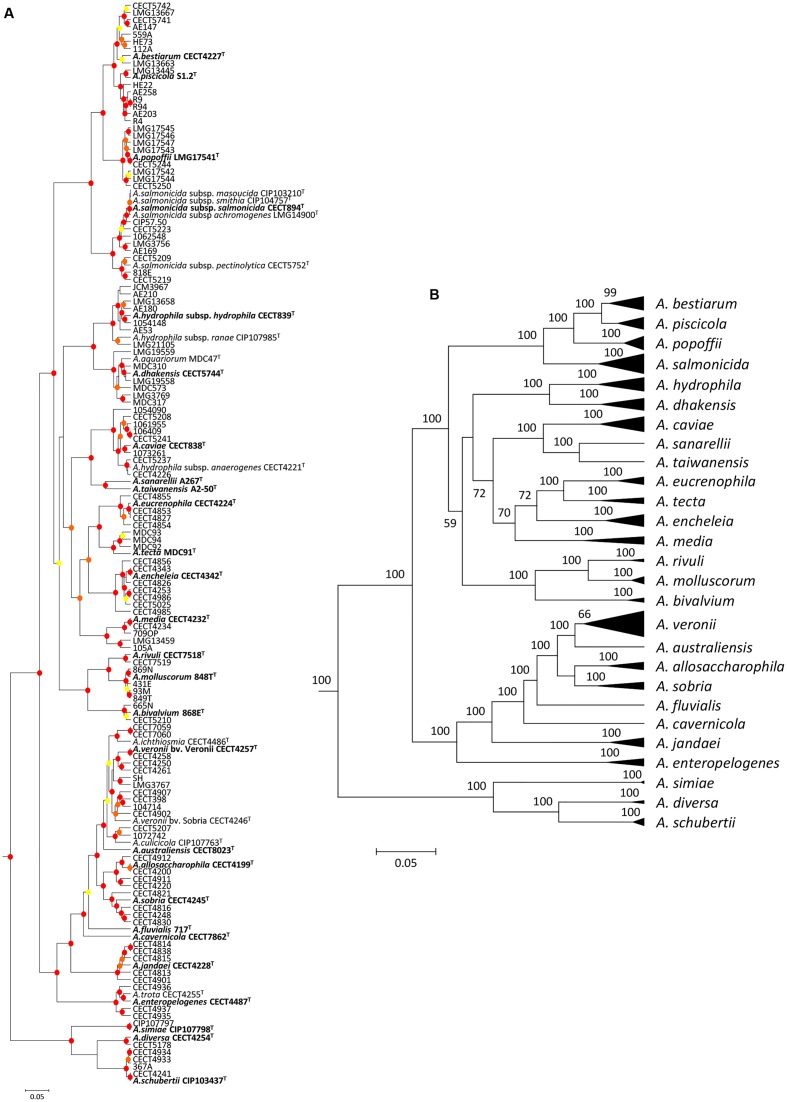
**Bayesian phylogenetic analysis of the genus *Aeromonas*.** **(A)** The phylogenetic tree shows the affiliation of the 150 *Aeromonas* strains. Posterior probability values > 50% are indicated at nodes by circles in yellow (50–70%), orange (70–90%), or red (90–100%). **(B)** Clusters of sequences belonging to the same species or species complex were collapsed in black triangles. Bayesian posterior probabilities are indicated in the nodes. Scale bar shows the number of substitutions per site.

The analysis involved 150 *Aeromonas* strains, in which we determined the full gene sequence of two housekeeping genes, malate dehydrogenase (*mdh*) and recombinase A (*recA*). The number of total positions analyzed was 2,007 bp. All positions containing gaps and missing data were eliminated in the construction of the phylogenetic tree. The best model selected for the concatenated sequences was the general time reversible (GTR) using a discrete gamma distribution and a fraction of invariable sites (GTR+G+I). **Figure 1** shows the *Aeromonas* Bayesian phylogeny with the posterior values obtained for each node, which were higher than 90% for the majority of clades. The figure also includes a collapsed tree (**Figure 1B**) to facilitate the visualization of the species distribution.

### Divergence Time Estimation

**FIGURE 2:**
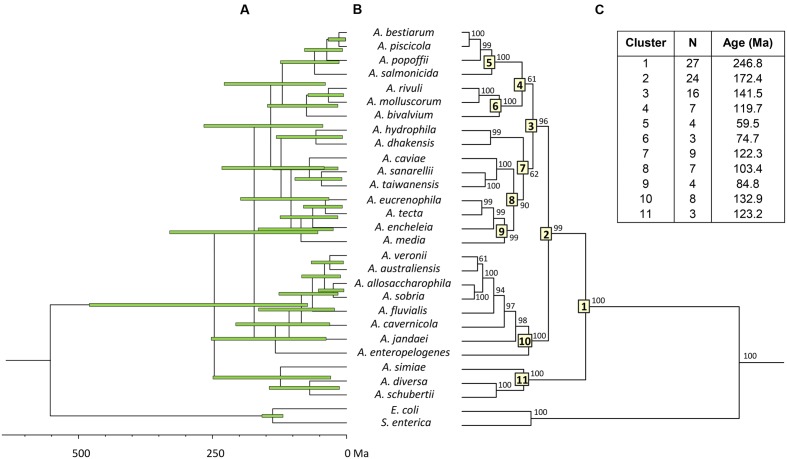
***Aeromonas* Bayesian consensus tree chronogram.** **(A)** Divergence time estimates for the *Aeromonas* species. Horizontal bars (green) indicate the 95% highest posterior density (HPD) values. Scale bar at the bottom represents divergence times in millions of years (Ma, Mega annum). **(B**) Bayesian posterior probability values (>50%) are shown at the nodes. Major *Aeromonas* species clades are indicated by framed numbers in the corresponding node. **(C)** Number of species (N), estimated ages for the genus *Aeromonas* (cluster 1), and the major clades of the chronogram.

**FIGURE 3:**
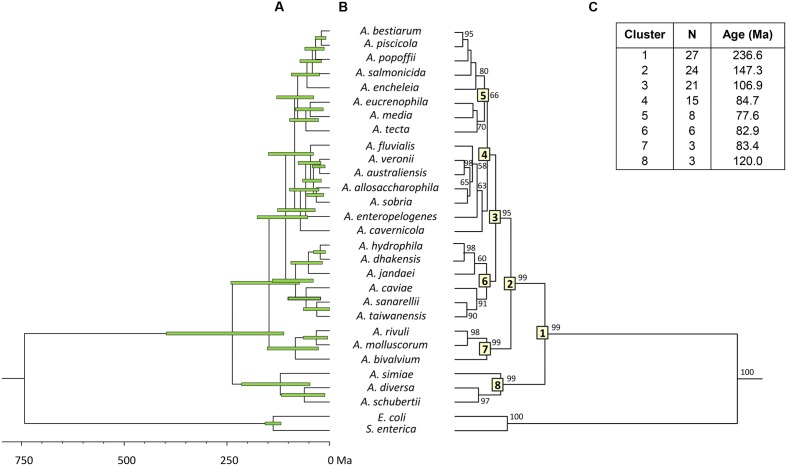
***Aeromonas* Bayesian species tree chronogram.** **(A)** Divergence time estimates for the *Aeromonas* species. Horizontal bars (green) indicate the 95% highest posterior density (HPD) values. Scale bar at the bottom represents divergence times in millions of years (Ma, Mega annum). **(B)** Bayesian posterior probability values (>50%) are shown at the nodes. Major *Aeromonas* species clades are indicated by framed numbers in the corresponding node. **(C)** Number of species (N), estimated ages for the genus *Aeromonas* (cluster 1), and the major clades of the chronogram.

To estimate the relative branching times, we used only one sequence for each species, because the inclusion of more sequences of the same species would artificially inflate the number of branching events toward the tip of the trees, producing misleading results (Fontaneto et al., 2012). We conducted this analysis using two different approaches, constructing the trees from either the consensus or the species sequences. The BEAST program was used to obtain Bayesian chronograms with the selected model of evolution, a relaxed molecular clock model and a calibration point of 140 Ma. **Figures 2** and **3** show the chronograms corresponding to the consensus and species tree, respectively. In both trees the main clades were well-statistically supported and exhibited quite a similar clade distribution, with the exception of the *A. veronii* group. Our estimates for the origin of the genus *Aeromonas* suggest that it began to diversify approximately 250 Ma ago (**Figures 2C** and **3C**).

### *Aeromonas* Diversification Rates

**FIGURE 4:**
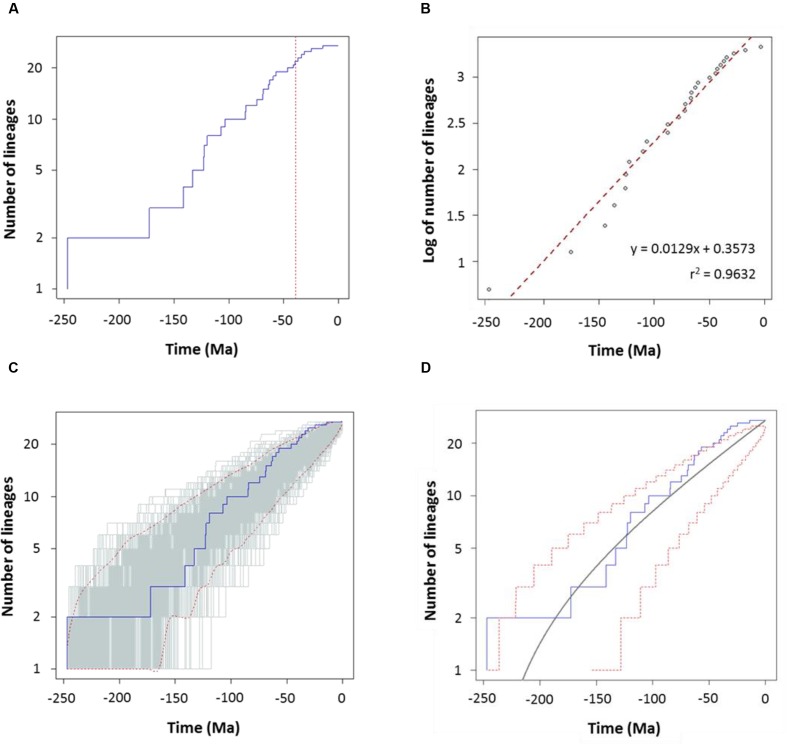
**Diversification analysis of the genus *Aeromonas* using the consensus chronogram.** **(A)** Empirical lineages-through-time (LTT) plot (blue line). Dotted red vertical line indicates the estimated rate shift (39 Ma). **(B)** Linear regression analysis of the LTT curve. Gray dots represent the empirical LTT plot. Red dashed line represents the best-fitting straight line through the points. **(C)** LTT plots obtained from 1,000 simulated phylogenies (gray lines) under a Yule process, compared with the empirical LTT curve (blue line). Red dashed lines show 95% confidence intervals. **(D)** Theoretical LTT plot (gray line) obtained with a constant diversification rate (λ = 0.0103) compared with the empirical LTT curve (blue line). Red dashed lines show 95% confidence intervals.

**FIGURE 5:**
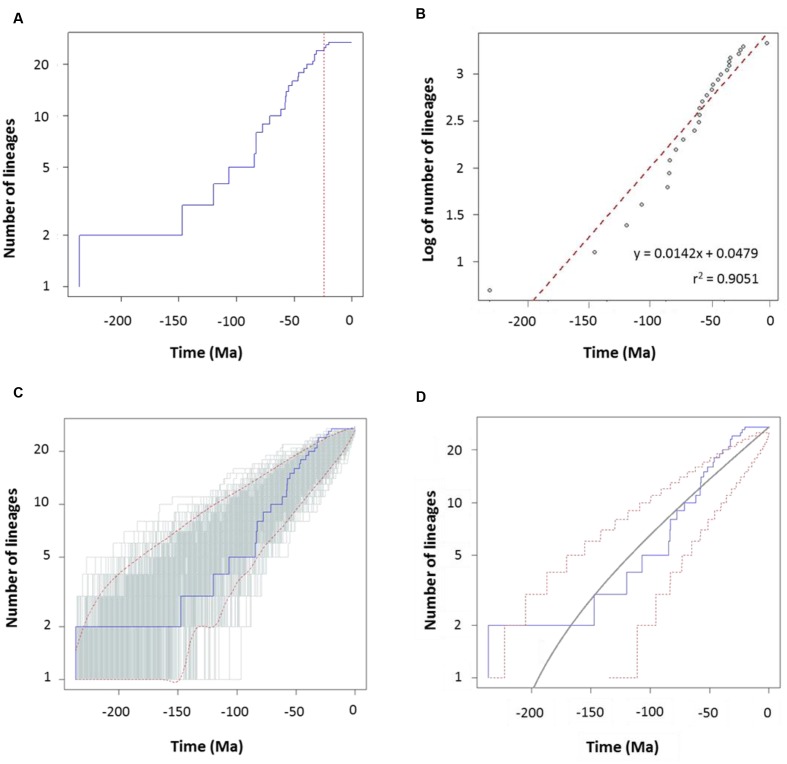
**Diversification analysis of the genus *Aeromonas* using the species tree chronogram.** **(A)** Empirical lineages-through-time (LTT) plot (blue line). Dotted red vertical line indicates the estimated rate shift (24.3 Ma). **(B)** Linear regression analysis of the LTT curve. Gray dots represent the empirical LTT plot. Red dashed line represents the best-fitting straight line through the points. **(C)** LTT plots obtained from 1,000 simulated phylogenies (gray lines) under a Yule process, compared with the empirical LTT curve (blue line). Red dashed lines show 95% confidence intervals. **(D)** Theoretical LTT plot (gray line) obtained with a constant diversification rate (λ = 0.0127) compared with the empirical LTT curve (blue line). Red dashed lines show 95% confidence intervals.

To characterize the clade diversification as a function of time, we determined the widely used expected number of lineages versus time (LTT plots) method. **Figures 4A** and **5A** show the semi-logarithmic LTT plots derived from the consensus and species chronograms, which revealed that the *Aeromonas* lineage accumulation through time roughly follows a straight line, suggesting a constant diversification rate. We calculated the diversification parameters from the chronograms using maximum likelihood and adjusted the data to a constant model of diversification. **Table 1** shows the diversification rates (λ) obtained from the chronograms analyzed. The diversification rate for the consensus approach was 0.0103 (*SE* = 0.0014), and for the species tree 0.0127 (*SE* = 0.0018). In both cases the extinction rate (μ) was near to 0.

**Table 1:** Diversification rate (λ) values obtained with different methods.

Chronogram	ML^a^	Linear model^c^	BI^d^

	**λ** +** SE^b^**	**λ (slope)**	**λ**
Consensus tree	0.0103 + 0.0014	0.0129	0.010–0.012
Species tree	0.0127 + 0.0018	0.0142	0.013


*^a^ML, Maximum Likelihood analysis.*

*^b^SE, standard error.*

*^c^See **Figures 4** and **5** for details.*

*^d^BI, Bayesian Inference method using the BAMM program.*

To corroborate the constancy of the diversification process in *Aeromonas*, we compared our LTT plots with those obtained from 1,000 simulated trees under a constant process of diversification, with the same size and diversification rate. **Figures 4C** and **5C** show that in both the consensus and species chronograms, the *Aeromonas* empirical LTT plot (blue line) lies within the range of the simulated phylogenies (gray lines). In addition, we obtained the theoretical LTT curve and the 95% confidence intervals around the predicted curve, as proposed by Paradis (2015), to infer the empirical LTT fit with a constant model of diversification. In the theoretical adjustment to our LTT plots, shown in **Figures 4D** and **5D**, although a few points above the theoretical curve fall outside the predicted intervals at the end of the process, in general a good fit was obtained.

To confirm if the diversification rate is really constant or has changed over time, we used maximum likelihood to fit the branching times derived from our chronograms to a variety of diversification models. As suggested by Rabosky (2006), we calculated the significance of ΔAIC_RC_ for the set of analyzed models by using the Yule model to simulate 5,000 phylogenies of the same size and diversification rate as those obtained from our data, and determined the *P* value from the resulting distributions. As can be seen in **Table 2**, in both cases (the consensus and the species tree) the null hypothesis of a Yule model should be rejected to a level of significance of α = 0.05, and the Yule 2-rates model accepted. This means that the diversification in *Aeromonas* is constant but with two different rates: λ_1_ = 0.0144 and λ_2_ = 0.0024 for the consensus (breakpoint at 39 Ma) and λ_1_ = 0.0175 and λ_2_ = 0.0030 for the species tree (breakpoint at 24.3 Ma). These data indicate a deceleration in the diversification rate at the end of the process, coinciding with approximately the last 40 Ma (vertical line in **Figures 4A** and **5A**).

**Table 2:** Fit of alternative diversity models to LTT plots derived from the consensus and species tree Bayesian chronograms.

	AIC^a^							ΔAIC_RC_ test^b^		
		
Chronogram	Yule	Birth–death	Best constant model	DDL	DDX	Yule 2-rates	Best variable model	ΔAIC_RC_	*P*-value^c^	Best model
Consensus tree	157.87	159.87	Yule	153.71	158.14	152.51	Yule 2-rates	5.3583	0.0311	Yule 2-rates
Species tree	147.60	149.60	Yule	144.70	149.10	140.99	Yule 2-rates	6.5999	0.0149	Yule 2-rates


*^a^Akaike Information Criterium.*

*^b^ΔAIC_RC_ test (Rabosky, 2006).*

*^c^*P*-value obtained by simulation (5,000 iterations). The null hypothesis of constant rate diversification (Yule) can be rejected at the *P*-value < 0.05.*

**FIGURE 6:**
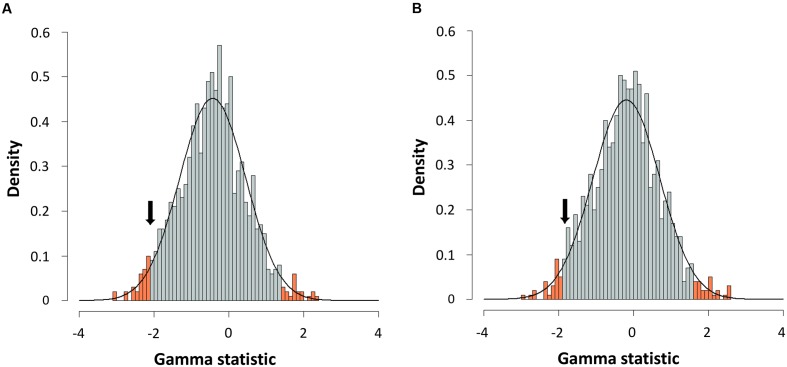
**Gamma statistic distribution.** Gamma statistic distribution obtained by simulating 1,000 phylogenies under the Yule model using a Bayesian approach from the consensus **(A)** or the species tree **(B)**. The arrows indicate the empirical gamma value (γ) obtained. Red bars show the 95% limits of distribution.

For further corroboration, we determined the gamma statistic of Pybus and Harvey, a powerful tool principally used for comparing models of decreasing speciation rate through time and a constant rate of diversification (Pybus and Harvey, 2000; Fordyce, 2010). We thus obtained an estimated γ value from both chronograms, with values of -2.1015 for the consensus and -1.8420 for the species tree. Although both γ values were negative, suggesting a possible deceleration through time, they were greater than those corresponding to critical values obtained by simulating 1,000 phylogenies under a constant rate model (Yule) at a level of α = 0.05 (**Figure 6**). Thus, a constant diversification rate cannot be rejected for our phylogenies.

**FIGURE 7:**
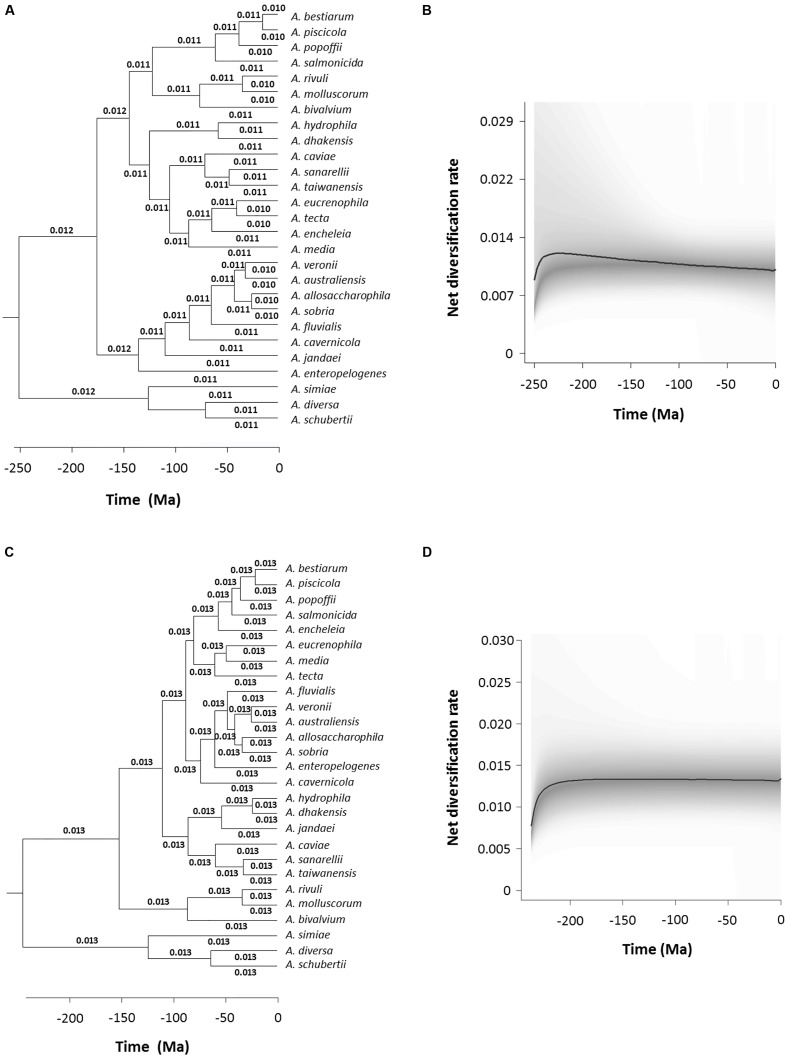
**Bayesian Analysis of Macroevolutionary Mixtures (BAMM) analysis.** Branch-specific diversification rates estimation based on the consensus **(A)** and species **(C)** trees. Net diversification rates-through-time plot for *Aeromonas* based on the consensus **(B)** and species **(D)** trees. Curved black lines represent the median values obtained and the 95% confidence intervals are shadowed in gray.

We performed an analysis to detect and quantify evolution rate heterogeneities with the BAMM program, which uses Bayesian inference to determine the estimated diversification rate for each branch in the phylogeny. **Figure 7A** shows a chronogram corresponding to the consensus sequences with the diversification rate values in each branch, which varied from 0.010 to 0.012. This result is also represented graphically in **Figure 7B**, which depicts the *Aeromonas* net diversification rate through time. In addition, we calculated the probability of no change in the diversification rate, which was 0.99, while the probability of a shift was 0.0086. Finally, we used the BAMM program to estimate the Bayes factor, a parameter that evaluates the probability of changes in the diversification rate, which in the case of the consensus chronogram was 114.942. A value higher than 100 is considered to provide decisive evidence of no change (**Table 3**). **Figures 7C,D** show the results obtained with BAMM from the species tree analysis. A diversification rate value of 0.013 was determined for all branches in the tree. Probabilities were 0.99 for no change and 0.0084 that a shift occurred, which are similar to the results achieved with the consensus sequences (**Table 3**). The Bayes factor in this case was 117.661.

**Table 3:** Diversification analyses.

Chronogram	LTT plot	Regression line (*r*^2^)	ΔAIC_RC_ test	Gamma statistic (γ)	BAMM analysis		
					
					0 shift	1 shift	Bayes factor
Consensus tree	constant	0.9632	Yule 2-rates	Yule	0.99	0.0086	114.9
Species tree	constant	0.9051	Yule 2-rates	Yule	0.99	0.0084	117.7


## Discussion

The estimation of diversification rate changes and the time they occurred is crucial for understanding the evolutionary patterns of taxa. In this field, the number of studies on prokaryotes is very low considering they represent the majority of the global biomass of living organisms and until recently dominated the history of life. The few studies in the literature about bacterial diversification (Martin et al., 2004; Vinuesa et al., 2005; Barraclough et al., 2009; Morlon et al., 2012; Lorén et al., 2014), are far less complete than those published on higher organisms. In the current work, we estimated the model and the speciation rate of *Aeromonas* based on phylogenetic reconstructions of evolutionary relationships. The work was performed using molecular data from the sequences of two housekeeping genes (*mdh* and *recA*) obtained from 150 strains belonging to 27 species of *Aeromonas*. When working with phylogenies that mix variation between and within species, it is often necessary to reduce the trees to obtain phylogenies with only one sequence per species. This is because the majority of methods used in diversification studies assume that the original tree is a phylogeny of species/monophyletic populations, rather than of specimen/strain/population samples, and the inclusion of more than one sequence per species could invalidate the results (Fontaneto et al., 2012).

The phylogeny constructed from the concatenated sequences corroborates the monophyletic origin of this group of bacteria. In the chronogram obtained, the majority of the nodes were strongly supported, with posterior values close to 100%. In addition, the main clade distribution was in agreement with previously published phylogenies (Martínez-Murcia et al., 2011; Roger et al., 2012; Colston et al., 2014; Lorén et al., 2014). We obtained a perfect clustering of the strains belonging to the same species, including those considered synonymous.

In eukaryotes, the fossil record provides an abundant source of temporal data, but information about the temporal dimension of prokaryote evolution is scarce, based on several indirect determinations and sometimes difficult to interpret. Limited information on specific metabolic groups of prokaryotes or data obtained from analyses of isotopic concentrations and detection of biomarkers – such as oxygen or the anaerobic formation of methane – have been used as indirect sources of calibrations for dating prokaryote phylogenies. Hence, it has been possible to constrain some nodes in the prokaryote timescale (Battistuzzi et al., 2004). In our study, we used only one indirect calibration point, due to the absence of more reliable calibration data, which can be a source of uncertainty, and may also explain the relatively large confidence intervals obtained (**Figure 2A**). The calibration point we have used for the divergence between *Escherichia* and *Salmonella*, 140 Ma (120–160 Ma), was proposed by Ochman and Wilson based on calibrated rates of ribosomal RNA divergence (Ochman and Wilson, 1987a). This calibration point was later validated by Retchless and Lawrence (2007), who analyzed ortholog genes present in three different *E. coli* and *S. enterica* genomes. They calculated the average divergence time for the entire genomes as well as for individual genes, determining an interval of 70 Ma for the divergence of *E. coli* and *S. enterica*, depending on the gene analyzed. Based on their results, considering in particular the *mdh* and *recA* gene sequences, the divergence between *E. coli* and *Salmonella* would have occurred 168 and 120 Ma ago, respectively. These data fully match the interval chosen in our analysis (160–120 Ma).

Both chronograms, the consensus (**Figure 2**; 246.8 Ma) and species (**Figure 3**; 236.6 Ma) trees, suggest that the divergence of the genus *Aeromonas* began approximately 250 Ma ago, between the Permian and Triassic periods. These results coincide with those of several bacterial genera such as *Mycoplasma. Rickettsia. Mycobacterium*, and *Streptococcus* (170–325 Ma) dated by Battistuzzi et al. (2004). In an attempt to establish a genomic timescale of prokaryote evolution, they determined the divergence time of the major groups of *Bacteria* and *Archaea* from a data set of 32 protein sequences (about 7,600 amino acids) common to 72 species. They used several calibrations based on geological events, the origin of *Cyanobacteria*, or fossil and molecular times of plant-animal divergence. The average divergence times obtained for the different eubacteria genera that include more than one species ranged between 36 (*Listeria*) and 1,061 Ma (*Clostridium*).

The results obtained with the LTT plots, the diversification models, and the gamma of Pybus and Harvey (**Table 3**) support the hypothesis of a constant cladogenesis in *Aeromonas* with no or an undetectable extinction rate. The diversification rate values were almost identical, varying between 0.010 and 0.0142 (**Table 1**) according to the method used for the analysis. These values are in complete agreement with those of Lorén et al. (2014), who determined the diversification rate of *Aeromonas* from the sequences of the type strains of this bacterial genus. Unfortunately, to our knowledge there are no more quantitative data about the diversification rates of other bacterial groups. Martin et al. (2004) analyzed a wide variety of prokaryotes to determine their diversification pattern, which in all cases proved to be constant, but without measuring the diversification rates. More recently, Morlon et al. (2012) investigated the diversification of *Borrelia burgdorferi sensu lato*, an intracellular pathogenic bacterium, from multilocus genomic sequence data. In this case, diversification was not constant, with explosive radiations followed by rapid decreases, but the rate was not calculated.

In an analysis of 163 phylogenies of animal taxa, McPeek and Brown (2007) determined their diversification rates, which ranged from 0.013 to 3 speciation events per million years. Similarly, Magallón and Sanderson (2001) established the diversification rate for angiosperms as a whole, which ranges from 0.077 (μ/λ = 0.9) to 0.089 (μ/λ = 0.0) net speciation events per million years. The *Aeromonas* λ values we determined coincide with the lower limit for the eukaryotes, being one or two orders of magnitude lower than those of the majority of animal and plant species.

To corroborate the goodness of our results, we used two different approaches for determining the model of cladogenesis in *Aeromonas* from the LTT plots. Firstly, phylogenies were simulated with a constant model of diversification and the parameters determined from our data, and the empirical LTT plot was then compared with those obtained by simulation. Secondly, the theoretical LTT curve was calculated as well as a prediction interval around the predicted curve, as proposed by Paradis (2015). Our results show that in both chronograms (consensus and species trees), the derived empirical LTT plots fit well with the theoretical curve, falling within the predicted intervals with only a few points outside at the end of the process. Nevertheless, as Paradis suggests, the presence of outliers could be related with the sample size or the λ-μ value.

After the testing of different models with constant and variable diversification rates, the Yule 2-rates was selected as the model with the best fit for our data: a diversification model with two different rates. In addition, according to a Bayesian analysis conducted with the BAMM program, *Aeromonas* followed a constant diversification rate model, although there is a small probability (0.0084 for the consensus and 0.0086 for the species tree) that a slight deceleration occurred in recent times. Nevertheless, as Hedges et al. (2015) suggest, this terminal drop in rate could be a normal characteristic of diversification plots related to their taxonomic level, because lower level clades are omitted. This pattern of constant diversification agrees with reports for most eukaryotes (Hedges et al., 2015) or a mammalian phylogeny including 4,510 present-day species (Stadler, 2011).

In summary, our results suggest that the diversification of *Aeromonas* began approximately 250 Ma ago, between the Permian and Triassic periods, when the number of higher organisms on earth increased considerably (Hedges et al., 2015). Since then, the process has remained constant through time, following a Yule model with a small probability of a deceleration during the last 40 Ma. The diversification rate values obtained were in complete agreement with those previously determined for the type strains of this genus using the sequences of five housekeeping genes (Lorén et al., 2014), although whenever possible the present analysis was performed with sequences from several strains of the same species.

## Author Contributions

MF, JL, and MCF conceived and designed research. AS and VA performed the experiments. AS, MF, and MCF analyzed the data. MF and MCF wrote the paper. JL made a substantial contribution to the analysis and interpretation of data and revised the final manuscript version. They all approved the final version to be published.

## Conflict of Interest Statement

The authors declare that the research was conducted in the absence of any commercial or financial relationships that could be construed as a potential conflict of interest.

## Footnotes

1 http://www.ncbi.nlm.nih.gov/genbank/

2 http://beast.bio.ed.ac.uk/logcombiner

3 http://beast.bio.ed.ac.uk/figtree

4 http://bamm-project.org
