# Supplementary material for: Evolutionary Roots and Diversification of the Genus Aeromonas
Source: Front Microbiol. 2017 Feb 8;8:127. doi: 10.3389/fmicb.2017.00127 (PMC5296313; doi:10.3389/fmicb.2017.00127)
Supplement: Supplementary file 1 [file Table_1.DOCX]

**Table S1.** *Aeromonas* strains and gene sequences used in this study.

| **Species / Complex^a^** | **Strain^b^** | **Other designation** | **Source / Geographical origin^c^** | **GenBank accession no.^d^** | |
| --- | --- | --- | --- | --- | --- |
|  |  |  |  | ***mdh*** | ***recA*** |
| *A. allosaccharophila* / *A. veronii* (n=5) | CECT4199^T^ | *A. veronii* | Diseased elvers of eel from a fish farm / Spain | HM163292 | KM260565 |
|  | CECT4200 |  | Diseased eel from a fish farm / Spain | KM507368 | KM260566 |
|  | CECT4220 |  | Faeces / USA | KM507369 | KM260567 |
|  | CECT4911 |  | Faeces / Switzerland | KM507370 | KM260568 |
|  | CECT4912 |  | Faeces / Switzerland | KM507371 | KM260569 |
| *A. australiensis* (n=1) | CECT8023^T^ |  | Irrigation water / Australia | KM507372 | KM260570 |
| *A. bestiarum* / *A. hydrophila* (n=9) | CECT4227^T^ |  | Diseased fish | HM163294 | KM260547 |
|  | 112A |  | Non-drinking water / Spain | JN660159 | KM260548 |
|  | 559A |  | Drinking water / Spain | JN660162 | KM260549 |
|  | AE147 |  | Lake water / Finland | JN660167 | KM260550 |
|  | CECT5741 |  | Environment / Germany | JN660175 | KM260551 |
|  | CECT5742 |  | Water / Switzerland | JN660176 | KM260552 |
|  | HE73 |  | Water from Iso-Kukka Lake / Finland | JN660179 | KM260553 |
|  | LMG13663 |  | Intestine of juvenile silver salmon | JN660183 | KM260554 |
|  | LMG13667 |  | Probably water / USA | JN660187 | KM260555 |
| *A. bivalvium* (n=3) | 868E^T^ |  | Cockle / Spain | HM163295 | KM260571 |
|  | 665N |  | Razor-shell / Spain | KM507373 | KM260572 |
|  | CECT5210 |  | Seawater / Spain | KM507413 | KM260619 |
| *A. cavernicola* (n=1) | CECT7862^T^ | *Aeromonas* sp. | Water of a brook in a cavern / Czech Republic | KM507374 | KM260573 |
| *A. caviae* (n=11) | CECT838^T^ |  | Epizootic of young guinea pigs | HM163296 | KM260574 |
|  | CECT4221^T^ | *A. hydrophila* subsp. *anaerogenes* | Used oil-emulsions | HM163304 | KM260575 |
|  | 106409 |  | Clinical, human / Mallorca, Spain | KM507375 | KM260576 |
|  | 1054090 |  | Clinical, human / Mallorca, Spain | KM507376 | KM260577 |
|  | 1061955 |  | Clinical, human / Mallorca, Spain | KM507377 | KM260578 |
|  | 1073261 |  | Clinical, human / Mallorca, Spain | KM507378 | KM260579 |
|  | CECT4226 |  | Oil emulsion | KM507379 | KM260580 |
|  | CECT5208 |  | Human abscess / Spain | KM507381 | KM260582 |
|  | CECT5237 |  | Human faeces / Spain | KM507382 | KM260583 |
|  | CECT5241 |  | Human faeces / Spain | KM507383 | KM260584 |
|  | LMG13459 |  | Infected fish | KM507384 | KM260585 |

| **Species / Complex^a^** | **Strain^b^** | **Other designation** | **Source / Geographical origin^c^** | **GenBank accession no.^d^** | |
| --- | --- | --- | --- | --- | --- |
|  |  |  |  | ***mdh*** | ***recA*** |
| *A. dhakensis / A. hydrophila* (n=9) | CECT5744^T^ | *A. hydrophila* subsp. *dhakensis* | Child with diarrhoea, faeces / Bangladesh | HM163305 | JN660346 |
|  | MDC47^T^ | *A. aquariorum* | Aquaria of ornamental fish / Portugal | HM163293 | JN660357 |
|  | LMG3769 |  | River water / India | JN660226 | JN660347 |
|  | LMG19558 |  | Child with diarrhoea, faeces / Bangladesh | JN660228 | JN660349 |
|  | LMG19559 |  | Child with diarrhoea, faeces / Bangladesh | JN660229 | JN660350 |
|  | MDC310 | *A. aquariorum* | Tropical fish / Portugal | JN660236 | JN660358 |
|  | MDC317 | *A. aquariorum* | Tropical fish / Portugal | JN660237 | JN660359 |
|  | MDC573 | *A. aquariorum* | Vega Baja hospital / Spain | JN660238 | JN660360 |
| *A. diversa* (n=2) | CECT4254^T^ |  | Human leg wound / USA | HM163298 | KM260586 |
|  | CECT5178 | *Aeromonas* sp. | Human leg wound / USA | KM507385 | KM260587 |
| *A. encheleia* (n=8) | CECT4342^T^ |  | Healthy European eels / Spain | HM163299 | KM260588 |
|  | CECT4253 | *Aeromonas* sp. HG11 | Human ankle suture / New Zealand | HM163300 | KM260589 |
|  | CECT4343 |  | Healthy glass eels / Spain | KM507386 | KM260590 |
|  | CECT4826 |  | Water from an artesian well / United Kingdom | KM507387 | KM260591 |
|  | CECT4856 |  | Water from Mohawk River / USA | KM507388 | KM260592 |
|  | CECT4985 |  | Hospital environment / Germany | KM507389 | KM260593 |
|  | CECT4986 |  | Drinking water well / Finland | KM507390 | KM260594 |
|  | CECT5025 |  | Drinking water well / Finland | KM507391 | KM260595 |
| *A. enteropelogenes* (n=5) | CECT4487^T^ |  | Human faeces / India | HM163301 | KM260596 |
|  | CECT4255^T^ | *A. trota* | Human stool / India | HM163325 | KM260597 |
|  | CECT4935 |  | Human appendix / USA | KM507392 | KM260598 |
|  | CECT4936 |  | Human stool / Thailand | KM507393 | KM260599 |
|  | CECT4937 |  | Human stool / Indonesia | KM507394 | KM260600 |
| *A. eucrenophila* (n=5) | CECT4224^T^ |  | Fresh water fish | HM163302 | KM260556 |
|  | CECT4827 |  | Carp, ascites | KM507395 | KM260557 |
|  | CECT4853 |  | Carp, ascites | KM507396 | KM260558 |
|  | CECT4854 |  | Urban well / Germany | KM507397 | KM260559 |
|  | CECT4855 |  | Rural well / Germany | KM507398 | KM260560 |
| *A. fluvialis* (n=1) | 717^T^ |  | Water from Muga River / Spain | HM163303 | KM260601 |

| **Species / Complex^a^** | **Strain^b^** | **Other designation** | **Source / Geographical origin^c^** | **GenBank accession no.^d^** | |
| --- | --- | --- | --- | --- | --- |
|  |  |  |  | ***mdh*** | ***recA*** |
| *A. hydrophila / A. hydrophila* (n=9) | CECT839^T^ | *A. hydrophila* subsp. *hydrophila* | Tin of milk with fishy odour | HM163306 | JN660318 |
|  | CIP107985^T^ | *A. hydrophila* subsp. *ranae* | Liver of a frog with septicaemia / Thailand | HM163307 | JN660345 |
|  | 1054148 |  | Clinical, human / Mallorca, Spain | JN660200 | JN660319 |
|  | AE53 |  | Lake water / Finland | JN660203 | JN660322 |
|  | AE180 |  | Lake water / Finland | JN660206 | JN660325 |
|  | AE210 |  | Lake water / Finland | JN660207 | JN660326 |
|  | JCM3967 | *A. hydrophila* subsp. *hydrophila* | NA | JN660217 | JN660336 |
|  | LMG13658 | *A. hydrophila* subsp. *hydrophila* | Faeces / Switzerland | JN660221 | JN660340 |
|  | LMG21105 | *A. hydrophila* subsp. *hydrophila* | Waste water lagoon / Morocco | JN660225 | JN660344 |
| *A. jandaei* (n=6) | CECT4228^T^ |  | Faeces from patient with diarrhea / USA | HM163309 | KM260604 |
|  | CECT4813 |  | Faeces | KM507401 | KM260605 |
|  | CECT4814 |  | NA | KM507402 | KM260606 |
|  | CECT4815 |  | Faeces | KM507403 | KM260607 |
|  | CECT4838 |  | Blood / USA | KM507404 | KM260608 |
|  | CECT4901 |  | Leg wound / USA | KM507405 | KM260609 |
| *A. media* (n=4) | CECT4232^T^ |  | Fish farm effluent | HM163310 | KM260610 |
|  | 105A |  | Non-drinking water / Spain | KM507406 | KM260611 |
|  | 709OP |  | Large oyster / Spain | KM507407 | KM260612 |
|  | CECT4234 |  | Fish farm pond | KM507408 | KM260613 |
| *A. molluscorum* (n=5) | 848T^T^ |  | Wedge-shell / Spain | HM163311 | KM260614 |
|  | 93M |  | Mussel / Spain | KM507409 | KM260615 |
|  | 431E |  | Cockle / Spain | KM507410 | KM260616 |
|  | 849T |  | Wedge-shell / Spain | KM507411 | KM260617 |
|  | 869N |  | Razor-shell / Spain | KM507412 | KM260618 |
| *A. piscicola* / *A. hydrophila* (n=8) | S1.2^T^ |  | Wild diseased Atlantic salmon / Spain | HM163312 | JN660307 |
|  | R4 |  | Diseased fish / Spain | JN660191 | JN660308 |
|  | R9 |  | Diseased fish / Spain | JN660192 | JN660309 |
|  | R94 |  | Diseased fish / Spain | JN660193 | JN660310 |
|  | AE203 |  | Lake water / Finland | JN660169 | JN660285 |
|  | AE258 |  | Lake water / Finland | JN660171 | JN660286 |
|  | HE22 |  | Water from Salajärvi Lake / Finland | JN660178 | JN660294 |
|  | LMG13445 | *A. bestiarum* | Human / Germany | JN660180 | JN660296 |

| **Species / Complex^a^** | **Strain^b^** | **Other designation** | **Source / Geographical origin^c^** | **GenBank accession no.^d^** | |
| --- | --- | --- | --- | --- | --- |
|  |  |  |  | ***mdh*** | ***recA*** |
| *A. popoffii / A. hydrophila* (n=9) | LMG17541^T^ |  | Drinking water production plant / Belgium | HM163313 | JN660311 |
|  | CECT5244 |  | Water from Noguera Pallaresa River / Spain | KM507414 | KM260620 |
|  | CECT5250 |  | Continental water / Spain | KM507415 | KM260621 |
|  | LMG17542 |  | Drinking water production plant / Belgium | JN660194 | JN660312 |
|  | LMG17543 |  | Drinking water production plant / Belgium | JN660195 | JN660313 |
|  | LMG17544 |  | Drinking water production plant / Belgium | JN660196 | JN660314 |
|  | LMG17545 |  | Drinking water production plant / Belgium | JN660197 | JN660315 |
|  | LMG17546 |  | Drinking water service reservoir / United Kingdom | JN660198 | JN660316 |
|  | LMG17547 |  | Drinking water treatment plant / United Kingdom | JN660199 | JN660317 |
| *A. rivuli* (n=2) | CECT7518^T^ |  | Water, karst region / Germany | JN215542 | KM260622 |
|  | CECT7519 |  | Water, karst region / Germany | KM507416 | KM260623 |
| *A. salmonicida* / *A. hydrophila* (n=13) | CECT894^T^ | *A. salmonicida* subsp. *salmonicida* | Atlantic salmon from Cletter River/United Kingdom | HM163317 | JN660361 |
|  | CECT5752^T^ | *A. salmonicida* subsp*. pectinolytica* | Water from a cistern / Spain | HM163316 | JN660399 |
|  | CIP103210^T^ | *A. salmonicida* subsp. *masoucida* | Sakuramasou, heart blood | HM163315 | JN660398 |
|  | CIP104757^T^ | *A. salmonicida* subsp. *smithia* | Roach, ulcer / United Kingdom | HM163318 | JN660400 |
|  | LMG14900^T^ | *A. salmonicida* subsp. *achromogenes* | Brown trout from Dee River / United Kingdom | HM163314 | JN660397 |
|  | 818E |  | Cockle / Spain | JN660249 | JN660372 |
|  | 1062548 |  | Clinical, human / Mallorca, Spain | JN660252 | JN660375 |
|  | AE169 |  | Seawater from Baltic Sea / Finland | JN660253 | JN660376 |
|  | CECT5209 | *A. salmonicida* subsp. *salmonicida* | Seawater / Spain | JN660258 | JN660381 |
|  | CECT5219 |  | Cake / Spain | JN660262 | JN660385 |
|  | CECT5223 |  | Mussel / Spain | JN660265 | JN660388 |
|  | CIP57.50 |  | Quality control strain | JN660271 | JN660394 |
|  | LMG3756 |  | Human, clinical | JN660272 | JN660395 |
| *A. sanarellii* (n=1) | A2-67^T^ |  | Clinical, wound / Taiwan | HM163319 | KM260624 |
| *A. schubertii* (n=5) | CIP103437^T^ |  | Human forehead abscess / USA | HM163320 | KM260625 |
|  | 367A |  | Aquaculture / South Africa | KM507417 | KM260626 |
|  | CECT4241 |  | Forehead abscess | KM507418 | KM260627 |
|  | CECT4933 |  | Leg wound of a 47 years old man | KM507419 | KM260628 |
|  | CECT4934 |  | Leg wound of a 36 years old woman | KM507420 | KM260629 |
| *A.simiae* (n=2) | CIP107798^T^ |  | Monkey faeces / France | HM163321 | KM260630 |
|  | CIP107797 |  | Monkey faeces / France | KM507421 | KM260631 |

| **Species / Complex^a^** | **Strain^b^** | **Other designation** | **Source / Geographical origin^c^** | **GenBank accession no.^d^** | |
| --- | --- | --- | --- | --- | --- |
|  |  |  |  | ***mdh*** | ***recA*** |
| *A. sobria* (n=5) | CECT4245^T^ |  | Carp / France | HM163322 | KM260632 |
|  | CECT4248 |  | Fish | KM507422 | KM260633 |
|  | CECT4250 |  | Fish | KM507423 | KM260634 |
|  | CECT4821 |  | Pike | KM507424 | KM260635 |
|  | CECT4830 |  | Fish | KM507425 | KM260636 |
| *A. taiwanensis* (n=1) | A2-50^T^ |  | Clinical, wound / Taiwan | HM163323 | KM260637 |
| *A. tecta* (n=4) | MDC91^T^ |  | Faecal sample of a 5 years old child / Switzerland | HM163324 | KM260561 |
|  | MDC92 |  | Surface swab of a rainbow trout | KM507426 | KM260562 |
|  | MDC93 |  | Tap water | KM507427 | KM260563 |
|  | MDC94 |  | Faecal sample of an asymptomatic adult | KM507428 | KM260564 |
| *A. veronii / A. veronii* (n=17) | CECT4257^T^ | *A. veronii* bv. Veronii | Sputum of drowning victim / USA | HM163327 | KM260638 |
|  | CECT4246^T^ | *A. veronii* bv. Sobria | Infected frog suffering from 'red leg' disease | HM163326 | KM260639 |
|  | CIP107763^T^ | *A. culicicola* | Mid gut of *Culex quinquifasciatus* / India | HM163297 | KM260640 |
|  | CECT4486^T^ | *A. ichthiosmia* | Surface water | HM163308 | KM260641 |
|  | 104714 |  | Clinical, human / Mallorca, Spain | KM507429 | KM260642 |
|  | 1072742 |  | Clinical, human / Mallorca, Spain | KM507430 | KM260643 |
|  | CECT398 |  | Human faeces of a child with diarrhoea / USA | KM507399 | KM260602 |
|  | CECT4258 |  | Diarrheic stool / USA | KM507431 | KM260644 |
|  | CECT4261 |  | Maxillary sinus / USA | KM507432 | KM260645 |
|  | CECT4816 | *Aeromonas* sp. | Burbot kidney | KM507433 | KM260646 |
|  | CECT4902 |  | Environment / Germany | KM507434 | KM260647 |
|  | CECT4907 |  | Faeces / Switzerland | KM507435 | KM260648 |
|  | CECT5207 |  | Human blood / Spain | KM507380 | KM260581 |
|  | CECT7059 |  | Drinking water supply / Spain | KM507436 | KM260649 |
|  | CECT7060 |  | Drinking water supply / Spain | KM507437 | KM260650 |
|  | LMG3767 |  | Human / India | KM507400 | KM260603 |
|  | SH | *A. culicicola* | Mid gut of *Culex quinquifasciatus* / India | KM507438 | KM260651 |

^a^ *Aeromonas* species and its associated complex; n, number of strains

^b^ T, type strain; CECT, Spanish Type Culture Collection; CIP, the Collection of Institut Pasteur; JCM, Japan Collection of Microorganisms; LMG, Belgian Co-ordinated Collections of Microorganisms

^c^ NA, not available

^d^ All sequences were determined by our research group
